# Nitrogen Nutrition of Fruit Trees to Reconcile Productivity and Environmental Concerns

**DOI:** 10.3390/plants7010004

**Published:** 2018-01-10

**Authors:** Corina Carranca, Gustavo Brunetto, Massimo Tagliavini

**Affiliations:** 1 Instituto Nacional de Investigação Agrária e Veterinária, Quinta do Marquês, Nova Oeiras, 2784-505 Oeiras, Portugal; 2 Departamento de Solos, Centro de Ciências Rurais, Universidade Federal de Santa Maria, Santa Maria 80576, Rio Grande do Sul, Brasil; brunetto.gustavo@gmail.com; 3 Faculty of Science and Technology, Free University of Bozen-Bolzano (UNIBZ), 39100 Bolzano, Italy; Massimo.Tagliavini@unibz.it

**Keywords:** cover crops, deciduous and evergreen fruit trees, internal N cycling, mineral and organic N fertilization, N losses, N uptake

## Abstract

Although perennial fruit crops represent 1% of global agricultural land, they are of a great economic importance in world trade and in the economy of many regions. The perennial woody nature of fruit trees, their physiological stages of growth, the root distribution pattern, and the presence of herbaceous vegetation in alleys make orchard systems efficient in the use and recycling of nitrogen (N). The present paper intends to review the existing literature on N nutrition of young and mature deciduous and evergreen fruit trees with special emphasis to temperate and Mediterranean climates. There are two major sources of N contributing to vegetative tree growth and reproduction: root N uptake and internal N cycling. Optimisation of the use of external and internal N sources is important for a sustainable fruit production, as N use efficiency by young and mature fruit trees is generally lower than 55% and losses of fertilizer N may occur with the consequent economic and environmental concern. Organic alternatives to mineral N fertilizer like the application of manure, compost, mulching, and cover crops are scarcely used in perennial fruit trees, in spite of the fact that society’s expectations call for more sustainable production techniques and the demand for organic fruits is increasing.

## 1. Introduction

There are increasing expectations from the society for the development of an ecologically-friendly management of mineral nutrition in agro-ecosystems. Several practices and approaches could be put in place to optimize the use of external as well as internal nitrogen (N) sources, including adapting rates of fertilizers to match tree needs, adopting highly efficient technology of nutrient supply (e.g., fertigation, organic fertilization) and splitting nutrient rates are among the means to improve external N use.

It is expected that in the 21st century, the adoption of genotypes efficient in the use of nutrients, able to either incorporate more N and/or to improve its assimilation in organic forms, will play a major role for increasing crop yield and secure food for an increasing world population [1], preserving non-renewable resources, like fertilizers, and maintaining the quality of soil. At least 60% of the world’s arable land suffers from mineral deficiencies or elemental toxicity problems [2,3,4,5]. Nutrient efficient crops are those that produce high yields per unit of applied or absorbed nutrient [3,6,7]. According to Srivastava [3], the perennial woody nature of fruit trees, their physiological stages of growth and the differential root distribution pattern make these plants more efficient on the use of nutrients than annual crops. Although fruit trees represent about 1% of the global agricultural land, they are of a great economic importance in many regions as well as in world trade [8]. Fruit trees, being perennial and allocating most of the net primary productivity to fruits, which contain relatively low N (Table 1), can be managed with relatively low N supply if the orchard is properly managed.

The present paper aims (i) at reviewing the existing literature on N nutrition of young and mature deciduous and evergreen fruit trees with special emphasis to temperate and Mediterranean climates; and (ii) providing recommendations for an environmentally sustainable orchard management.

## 2. Tree N Uptake and Internal Cycling

Nitrogen is often regarded as the most important mineral nutrient, limiting crop production in many agricultural crops worldwide. It has a major effect on crop yield and quality. It is a component of enzymes, vitamins, the chlorophyll molecule, and is involved in nucleic and amino acid synthesis and protein production. It is important for cell division and growth of young tissues (e.g., buds, flowers, leaves, twigs). Nitrogen also affects the absorption and distribution of practically all other nutrients in the plant, and is particularly important to the tree during flowering and fruit set [9,10,11,12,13].

Increasing soil and plant N availabilities enhance tree growth and vigour, two characteristics that, if managed with care are of the outmost importance for the economic success of newly planted fruit trees are therefore able to maximize their yield potential [11]. Too much vigour can be a result of excessive soil N availability and may compromise the onset of bearing, or even the yield, in the first years. Sometimes, even when the nutrient availability is lower than the lowest threshold, trees do not respond to fertilization because of adequate nutrient reserves built up in perennial organs in previous years [9,10,11,13,14]. Under limited available N, tree growth may appear normal but under sized. Nitrogen deficient trees carry a low fruit load and are highly erratic in their fruit bearing habit [12]. Moreover, they flush irregularly and produce short twigs and a reduced number of pale-green leaves.

Optimal concentration of N in fruits allows a proper development of skin colour, fruit size, and flavour. In fleshy fruits, fruit N concentration is higher during the first stage of fruit development (cytokinesis) and decreases thereafter, during the fruit growth until maturity due to both a lower uptake rate and to a dilution effect. Fruit N concentration in deciduous trees depends on many factors, including grafting combination (cultivar and rootstock), environmental conditions and orchard management, and generally vary from 2.5 to 9.8 g N kg^−1^ fruit dry weight [15,16].

Fruit trees, like most trees, use two main sources of N for their vegetative growth and reproduction: the root N uptake and the internal N cycling. The N available for root uptake can derive from mineral fertilizers or from mineralization of native N. Plant roots are able to absorb N in the nitrate (NO_3_^−^) as well as in the ammonium (NH_4_^+^) form, but in well-aerated soils NO_3_^−^ is the predominant N source [17]. Apple trees exposed to NH_4_^+^ formed many more flowers than NO_3_^−^-fed trees even when the NH_4_^+^ was available for only a very short period [17]. Throughout the experimental period, NH_4_^+^ fertilization led to higher values of the asparagine/arginine ratio in the tree than did NO_3_^−^ nutrition [17] demonstrating that asparagine is the main translocation compound for N. Arginine and, to a lesser degree, asparagine, were by far the most abundant levels of the soluble amino compounds in the fertilized trees far above those in the unfertilized trees [17]. If, and to what extent, the roots of fruit trees are able to absorb organic N (e.g., in the form of aminoacids [18] is still an open question. Recent evidence suggests that not only ectomycorrhizal fungi, but also arbuscular mycorrhizal associated with tree roots [19] expand the ability of trees to take up organic N.

After being taken up, the N is translocated through the xylem and allocated to the different organs. Seasonal changes in the amino acidic composition of the xylem sap composition of fruit trees (e.g., apple, cherry, walnut, grape) have been documented and allowed to distinguish between recently root absorbed N and the N deriving from winter storage [19,20].

A considerable amount of the nutrients translocated to the roots can be reloaded into the xylem and translocated back to the shoot, i.e., they are recycled within the plant [20,21,22]. It has been demonstrated for annual plant species that part of the N in the xylem represents a recycled fraction, there are few data for tree species because of the difficulty of measuring this process [21]. Although the contribution of recycled N to total N flux in the xylem is negligible during the period of spring remobilization of stored N, Grassi et al. [21] found that it increases exponentially after that period. Grassi et al. [21] observed in cherry trees that three months after bud burst about 45–50% of total N that passed through xylem was apparently derived from shoot-to-root recycling. They also stated that the recycled N in the xylem was inversely related to total N status of the tree. Therefore, the regulation of N uptake by roots involves shoot-to-root cycling of N: when trees are growing rapidly (e.g., the juvenile and transition phases), or when environmental conditions are more favourable for root activity, there is a high shoot demand for N and a lower proportion of N is translocated back to the roots as aminoacids. On the contrary, when the shoot demand for N is low, there is a relative increase of cycling N in the phloem, which, in turn, depresses further root N uptake.

Reserves throughout woody plants are important for several reasons. Winter survival depends on these adequate reserves. Although regulation of mobilization of root reserves remains unclear, both gibberellins and auxins are possibly involved [22]. If woody plants use root reserves early in the season, before bud break, most carbohydrates are translocated acropetally within the phloem during the growing season. Phloem loading is an active temperature-dependent process [22].

Nitrogen in young tissues, e.g., shoot tips, buds, and new leaves is present mostly as protein [12]; as new cells are formed, part of the protein N moves from older cells to newer ones, especially when the total N content of the plant is low. Young leaves increase their organic N until they have reached the maturity and full expansion. Under N deficiency, proteins are hydrolyzed (proteolysis) and the resulting aminoacids are distributed to the younger leaves and tips [23]. The proteolysis results in a collapse of the chloroplasts and a decline of the chlorophyll content. This is the reason why symptoms of N deficiency (yellowish colour) occur first in the older leaves.

^15^N labelled fertilizers have often been used in experiments to elucidate the fate of either the stores N or the fertilizer N. Nitrogen reserves are built up in the previous year and are used to support early growth in the following spring. The contribution of remobilised N to total N needs by trees depends on the tree age and size and the amount of stored N. In deciduous trees (e.g., pear, apple), internal N cycling comprises an intense withdrawal of N from leaves during senescence and its translocation to perennial organs (trunk, old stems, old roots) where they are stored [9,13,21]. In peach trees, for instance, about 50% of leaf N (about 30 kg N ha^−1^, according to Niederholzer et al. [24] is withdrawn before leaf abscission and translocated to storage organs. Roots of fruit trees will store large amounts of N during the winter months and are considered important sources of N in the spring [25]. Spring N remobilisation from storage organs to developing organs normally precedes root N uptake.

There is a substantial body of literature describing the N uptake and internal cycling of N in deciduous fruit trees, but less information is available for evergreen trees [10,26,27]. In evergreen trees (e.g., citrus), leaves are an important additional sink of N during the winter [10,26,28]. Similarly to deciduous trees, remobilisation of internal N reserves in evergreen trees are crucial for optimal shoot growth, flowering, and fruit set since bud break occurs when conditions (end on winter) are not always optimal for root N uptake [10,26,29,30]. Once N is absorbed by roots or remobilised from storage reserves, it is allocated to the organs that are developing according to their needs. Shoots, followed by fruits are the main N sinks in orange trees, whose uptake rates in Mediterranean districts in the Northern hemisphere is rather constant from April to November, but relatively less N is partitioned to fruits when the fertilizer N is supplied late in the season [28]. In several species, there is a low recovery of the labelled fertilizer N in the roots, trunk, and branches that, however, increase their fertilizer N content in winter probably due to the annual N recycling.

## 3. Nitrogen Fertilization of Fruit Trees

### 3.1. Nitrogen Needs

Fertilization is considered one of the most effective practices to increase the profits in fruit trees. Many studies report that N supply rates are often much higher than the tree needs: on one hand, the latter are often overestimated, especially in mature orchards; on the other hand, growers believe that due to relatively low fertilizer N use efficiency (FNUE), they have to apply N amounts much higher than those absorbed.

Nitrogen uptake needs by fruit trees can be predicted at yearly basis under field conditions by estimating the amounts of N in the fruits annually removed, the stored N, and N present in the abscised leaves. In mature trees, regularly pruned, and with a relatively stable framework’s biomass, N uptake needs can be estimated by considering the N amounts in the fruits, in the abscised leaves and in the pruning material [31,32] (Table 1). A meaningful assessment of N uptake needs for young trees should additionally take into account the N yearly allocated to perennial organs that significantly increase their size in the first years after transplanting.

**Figure 1:**
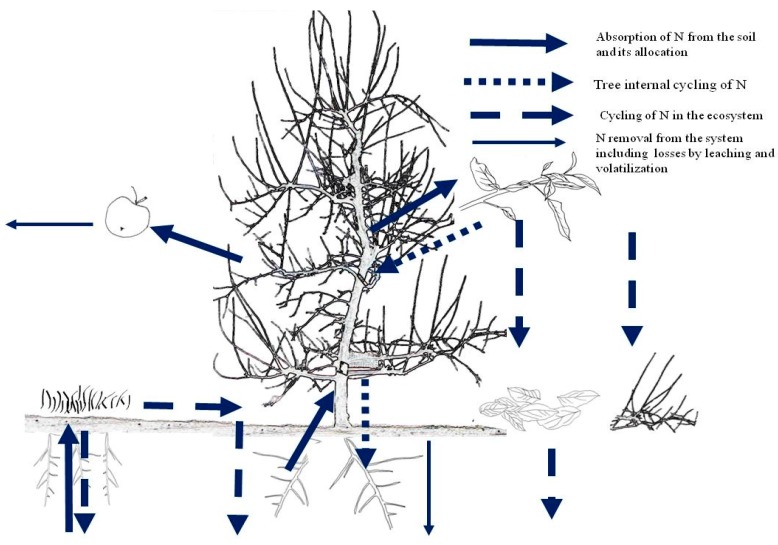
Nitrogen dynamics in a fruit tree ecosystem (Source: [32]).

As the soil may provide a significant fraction of the tree N needs, the fertilizer N rates should only supply part of the tree N needs. In general, N supply in orchards is carried out when the soil cannot provide a sufficient amount of N to feed the plant for obtaining the maximum performance. To decide about the amount of N to be supplied we can use either a budget at (i) orchard level or (ii) at soil level. The N dynamics in a fruit tree ecosystem is shown in Figure 1. Mass balance at orchard level considers N inputs (atmospheric N fixation, N depositions, N in irrigation waters) and outputs (N contained in the fruits and pruned material) at ecosystem level in order to maintain the soil fertility over time. Mass balance at soil level is often preferable for fine tuning the N supply rates to soil N availability. In this case, soil N availability by mineralisation of organic matter is also considered among the inputs, which are subtracted to the total N needs [33]. The resulting N supply rates might be then fine-tuned by considering the length of new shoots, the leaf N concentration, and chlorophyll level [10,11,15].

Trees subjected to excess N application can be more susceptible to disease attack (e.g., fire blight) and show premature fruit fall. Under N excess, fruit size might also be reduced and quality may deteriorate. In deficient plants, leaves are small, narrow and pale green, and shoots are shorten with a reduced number of laterals. Nitrogen deficiency first appears in older leaves which turn into a yellowish orange to purplish colour and drop prematurely [12]. As a result of a reduced shoot growth, N deficient trees have fewer buds and blossom and fruit set are, therefore, lower than N-sufficient trees [12]. The fruit peel tends to be smooth and pale in colour, and the juice will contain less soluble solids and acid concentration.

In view of the increasing global market, it is now strategic to improve fruit yield and quality offering a product that meets the standards for fresh market, storage or processing, therefore, fertilization should be adjusted to the final fruit destination [15].

### 3.2. Nitrogen Management in Young Orchards

In young fruit trees, N should never be limited, otherwise tree growth and a rapid achievement of final tree size will be impaired. Young N-deficient trees have less primary and lateral shoots. In citrus, all three shoot growth flushes, in March, June, and August, maybe impaired if N is sub-optimal [10]. Juvenile, non-fruiting trees have to be fertilized more frequently than fruit-bearing trees, using smaller amounts of nutrient at each application. The supply must also be targeted to the limited soil volume, where roots are present. Young non-bearing trees are highly dependent on new inputs of N, although the recovery of fertilizer N in the tree is relatively small during the first three years after transplant. In a trial carried out in Portugal, young non-bearing pear trees were regularly fertilized with N across the entire growing season, but in the first two months after the bud break the N uptake was very low and trees depended mostly on an intense remobilization of stored N at this period. Once remobilization ceased, root uptake provided the additional N used for seasonal growth. Nitrogen uptake was minimum at bud break but peaked in June/July, remaining more or less constant until the leaf fall [11]. Neto et al. [11] estimated a low FNUE in young pear trees, rising from 6% to 14% and 33%, respectively, in the first, second, and third year of growth, with an annual mineral N application of 6 g N tree^−1^. Unrecovered N in the soil-plant system was as large as 89%, 46%, and 53%, respectively in the first, second, and third year. A similar fertilizer N recovery was found by Menino et al. [10] in young orange trees. Under the field condition in a Mediterranean region Menino et al. [10] determined the N partitioning in young orange trees (Table 2).

Estimated tree N requirements over the first years after planting are related to the increase in the trunk cross-sectional area, therefore growth-based models may help to predict N needs of deciduous trees and should be included in decision support systems for fertilizer N recommendations [11].

### 3.3. Nitrogen Management of Mature Fruit Trees

Unlike immature trees, the N contents and biomass of the perennial parts of mature trees remain essentially constant from year-to-year. In these mature fruit trees, total tree N uptake reflects the amount of nutrient removed in harvested fruits (Table 1), abscised fruitlets and flowers, senescent leaves, pruning wood, and root turnover [10]. For practical reasons, it is useful to refer to the net amount of N removed per unit of produce. Then, in a mature orchard the net amount of removed N is equal to the amount of N immobilized in the skeleton and N in the fruits and pruning wood. Assuming that the soil N fertility is adequate, the fertilizer N rate to be applied to a mature orchard in a given year can be estimated by multiplying the value in Table 1 and the corresponding expected crop yield. For instance, in an apple orchard with an expected production of 60 T fruits ha^−1^, the corresponding amount of fertilizer N to be supplied to the trees is 60 × 0.9 = 54 kg N ha^−1^; in a peach orchard producing about 30 T fruits ha^−1^, the corresponding amount of fertilizer N to be applied is 30 × 2.7= 81 kg N ha^−1^. If we assume a 50% FNUE by the apple tree, an estimated fertilizer N rate of 108 kg N ha^−1^ (54 × 2 kg N ha^−1^) should be supplied. In a less fertile soil, for instance, when N supplied in the previous year(s) was insufficient, or in case of sandy soils with a low soil organic matter content, or after the establishment of a permanent vegetation cover in the orchard, the above recommended fertilizer N rates should be raised; on the contrary, it should be lowered if soil N is highly available.

Nitrogen efficient crops are important for maintaining or improving crop yield in the future. However, the few studies that have reported the recovery of applied fertilizer N by mature fruit trees grown in the field condition found values varying between 25% and 55% at a yearly time scale [7,34,35,36], even when the annual rate of fertilizer N was split into several applications [28]. The efficiency of fertilizer N recovery is influenced by soil properties (texture, organic matter), climate conditions, cultural practices (fertilization, irrigation), plant genotype (cultivar, root density), and rootstock. Under high levels of available soil N, the FNUE is normally low: for instance, the percent recovery of fertilizer N decreased by 40% moving from low to high N supply rates in orange trees [34].

A relatively low recovery of fertilizer N in the trees in the year of its application does not necessary mean that the remaining N is lost; on the contrary, a major challenge we now face in orchards concerns the ability of the soil-vegetation system to prevent the residual N from being lost. As orchards are semi-natural cropping systems, it is of outmost importance to enhance the FNUE in the medium- to long-term. Techniques that favour long-term maintenance of the residual N in the system should be promoted, by allowing herbaceous vegetation to take it up in autumn and winter or favouring its immobilization soil by microorganisms during the decomposition of the soil organic matter. These practices will reduce the amount of N prone to be lost by leaching, runoff, or volatilization [7,37].

A programme of fertilizer N supply is yearly needed to ensure the long-term productivity of trees by replenishing soil N. In mature deciduous trees, N is usually split into several applications during the growing season, in periods of active root and before sensitive periods when tree N should not be deficient (e.g., flowering, rapid shoot, and fruit growth). A variety of recommendations derive from the literature. For instance, N application is recommended since the beginning of bud break until six weeks after full bloom in fruit-bearing pear trees [38,39,40], whereas longer periods of N supply with smaller single rates were suggested by Quartieri et al. and Raese [41,42] on the basis of their results in order to improve tree N reserves. For mature citrus trees, addition of N at bud burst/start of flowering usually promotes the early growth of new plant tissues and maximize the tree productivity; between fruit set and until fruitlets have about 30 mm size, N should be added in reduced amounts to maintain vegetative growth and enhance fruit set; at fruit enlargement/maturity, reduced N amounts help achieve high fruit sugar content and optimal skin thickness and fruit acidity. In this phase, excess N may lead to rots and poor fruit quality. During the post-harvest phase, the addition of low amounts of nutrient will boost N reserves for the early growth in the next season but might prolong the leaf senescence and depress wood hardening.

## 4. Soil Management to Enhance Fertilizer Nitrogen Use Efficiency

Enhancing the ability of the soil to support the N nutrition is a key aspect for sustainable N management in orchards; this concept is especially important in organic farming, a type of agriculture that is gaining more and more importance both in terms of cultivated areas and consumers’ acceptance. Most soil N is organic N form and undergoes several chemical and biological transformations whose complete analysis goes beyond the scope of this review.

Several orchard management practices are useful to build-up soil organic N pools, as well as enhance the soil conditions that favour mineralisation. They include (Table 3) the supply of a large array of organic materials (compost, manure) for both tree nutritional function and to improve soil physical properties, cycling of crop residues (leaves, pruning material), the presence of a natural herbaceous vegetation in the orchard alleys as well as along the tree rows, the use of cover crops, especially those belonging to the Fabaceae family along the tree alleys, subsequently ploughed into the soil.

After its incorporation into the soil, fresh manure releases part of its N quite rapidly, due to the presence of inorganic N (NH_4_^+^, NO_3_^−^), urea, and peptide N fractions. The regular application of manure usually increases the soil organic matter and the activity of the microbial population, as well as the soil aggregation and soil hydraulic properties [43,44]. In addition to the environmental conditions, the rate of decomposition of the organic material depends on the quantity and composition of material, like its content on lignin and cellulose, and on the frequency of application [45,46]. If carefully managed, losses of N from manure due to leaching, soil erosion, volatilization, and denitrification can be reduced to the minimum.

Mulch has been used in orchards to retain moisture in the soil, suppress weeds and improve the soil fertility. Mulch consists of any type of material spread on the soil surface as a covering. There are several types of organic mulches, including the composted manure and other composted organic materials (bark, straw, senescent leaves). Newly-planted fruit trees under organic farming system should be mulched annually for the first 3–4 years to preserve soil moisture and reduce competition from weeds. Organic mulches have high carbon/nitrogen (C/N) ratio and contain very few available nutrients, including N. For this reason, these organic mulches can trap the residual N present in the soil and prevent its losses.

Cover crops in orchards may also improve plants productivity and simultaneously maintain the ecosystem functioning. Whenever water supply is not limiting, their presence in modern orchards have positive effects on crop yield by suppressing the weeds, controlling the soil erosion, improving the soil quality, and controlling the plant diseases and pests [47]. Cover crops are usually grasses or legumes, sown or spontaneous, but may include other herbaceous vegetation. Farmers are reluctant to use cover crops in young orchards due to the competition they will establish with the young trees, which may be detrimental to tree growth and future yields. Then, the choice of most suitable cover crop species must be based on well-adapted species to the region taking into account the establishment cost, weed suppression potential, the level of competition with trees, and the ability to improve soil quality. Legumes used as cover crops are important sources of external N, and their use is especially exploited under organic farming. Root nodules infected by rhizobia spp. fix and reduce atmospheric N_2_ into organic compounds and release it when nodules decompose in the soil. In addition, the residues of legume plants enhance the soil N availability for the main crop, by making some N deriving from the atmosphere available for the decomposition process. An oversupply of N to fruit trees can occur with legume cover crops. However, through proper management practices that suppress the growth and/or decomposition of the legume cover crop (such as mowing or planting non-legumes associated with the legumes to compete with them for N) the N supply to the tree roots will be limited. Symbiotic N_2_ fixation, however, has rarely been quantified in organically managed orchards. Abscised tree leaves and pruning wood contain significant N amounts. Abscised leaves undergo a series of simultaneous processes of mineralization and immobilization that make N available again for tree root uptake or maintain N in the soil microbial biomass [48]. These plant residues act as slow-release fertilizers since residues have to be fractionated first, and then mineralized before N is available for plant nutrition. Mineralization rate depends on the amount and composition of senescent leaves and pruning wood material, especially the long C chains, but also the soil type and climatic conditions.

The recycling of N, as a result of the decomposition of senescent leaves in soil was addressed in few studies with apple, peach, and pear trees [48,49,50,51]. The lifetime of fallen leaves in an orchard and the amount of N returned to the soil are important when analysing the N balance for a sustainable orchard management [48]. Nitrogen added to the soil by the fallen leaves increases with the amount of abscised leaves, and is affected by tree age and leaf area index (LAI) of trees [48]. Abscised pear leaves with a C/N ratio of 28 had a decomposition rate (*k*) varying from *k* = 0.0025 day^−1^ (d^−1^) to *k* = 0.0047 d^−1^ [48], while apple leaves with a C/N of 35, after having immobilised some N in the spring following their abscission, decomposed with a *k* = 0.0017 d^−1^ during a period of 102 weeks [50].

Pruning wood removed in winter has a high C/N ratio and high lignin content, but contains less N than abscised leaves. If the woody material remains on the soil surface or is incorporated into the soil, it is decomposed very slowly and its N release rate is low and not easy to be predicted. Pruning wood, especially if reduced to small size before soil addition, can trap the residual N present in the soil by the immobilisation process [52] and prevents the N losses.

## 5. Efficient Nitrogen Supply Methods

In rain-fed orchards, N should be supplied either before the rainy events or incorporated into the soil by tillage. If mineral fertilizer is used, more than one single dose should be applied along the season. Organic fertilizers can, instead, be applied at a single yearly dose, even in autumn or in winter. Foliar application could also be recommended to complement soil supply whenever trees need N and if it does not rain.

Whenever irrigation water is available, fertigation, i.e., the application of nutrients to fruit trees through the irrigation water, is a valuable tool for providing N whenever it is needed. Fertigation with drip emitters is the most efficient system to simultaneously distribute water and nutrients in small amounts throughout the growth season. This technique has shown good responses on tree growth, yield, and quality, and resulted in a uniform distribution pattern of applied water and N within the active root zone. Several studies have demonstrated a higher N use efficiency when N was supplied by fertigation compared to broadcast applications in apple [53], citrus [54], peach [55], sweet cherry [56], etc. Nitrogen losses by leaching under fertigation and drip irrigation depends on how water supply is managed: if water supply exceeds the maximum soil water holding capacity, water, and possibly N, is lost by deep percolation; if N is supplied at low rates, the risk of N losses is rather limited.

Foliar nutrient supply is often adopted in fruit trees to complement soil N supply. Fertilizers dissolved in water are sprayed to the aerial organs and absorbed by stomata, aqueous pores and, when the molecule is not-polar, by the cuticle itself. This technique cannot replace the soil N application of fertilizer, but it is a useful tool as particularly summarized in Table 4. Urea (with low content of biuret <0.3%) is the mostly used and cheapest foliar N fertilizer; due to its not-polar features, it is absorbed rapidly and with high efficiency (up to 90%, [57,58]. Suggested rates vary from 0.5% to 2%, but in autumn higher concentration (e.g., 5%) can be applied as well. If compared with soil N supply in late summer/early autumn, foliar urea supply improves tree N reserves without enhancing the risks of causing N losses by leaching. The urea-N that is not absorbed by the spayed leaves is likely to remain on the leaf surface, and when leaves abscise urea-N promotes rapid leaf decomposition on the soil. Dong et al. [59] found no significant differences in yield and fruit quality between soil and foliar N applications, but soil application increased the NO_3_^−^ leaching losses below the root zone, especially late in the growing season of apple trees. In the long run, it might also be risky fully shifting N supply from roots to leaves, as the former need to find adequate N levels in the soil to support their growth.

## 6. Future Directions

Modern agricultural management techniques in fruit orchards, including fertilization, need to reconcile economic and ecological issues, taking into particular attention the climate change adaptation practices. Soil, climate, management, and plant genotype especially influence the fertilizer N recovery; therefore, a better understanding of nutrients requirements by young and bearing fruit trees is necessary for an efficient yield of high-quality fruits. Proper rates and timings of fertilizer N to fit the tree’s N demand, in association with correct watering scheduling and pruning, should be assumed as a powerful tool for manipulating the delicate balance between vegetative and reproductive growth of fruit trees, then contributing to increase the fertilizer N use efficiency while minimizing N losses from the ecosystem. Simultaneously, the growing demand for organic fruits should stimulate the adoption of environmental-friendly practices that promote the increase of soil organic N, e.g., by mulching and cover crops, and take advantage of the presence of legumes able to fix atmospheric N_2_.

## Figures and Tables

**Table 1:** Indicative values of net N removal in mature trees (immobilized N in the skeleton, in the fruits and in the pruning wood) per fruit yield. The data refer to crop situations in which the production charge was high and the relationship between fruit production and vegetative activity is balanced (Source: [32]).

Tree Species	Net N Removal (g N/kg Fresh Fruit)
Apple	0.9
Peach	2.7
Pear	1.7
Orange	3.7
Walnut	10
Olive	22
Kiwi	4.5

**Table 2:** Source and age of nitrogen (N) present in the different organs of young ‘Lane Late’ orange trees at the end of the third year after transplant (Source: [10]).

Organ	N Content (g/Tree)			
	Total N in the Organ	Ndff of the Year	Ndff of Previous Year	N from Other Sources
New leaves	29.2	10.8	3.9	14.5
Old leaves	3.2	0.8	0.6	1.8
New branches	7.3	2.9	1.0	3.4
Old branches	7.2	2.8	1.2	3.2
Trunk	4.0	1.2	0.9	1.9
Fine roots	4.6	2.0	0.8	1.8
Old roots	12.3	3.5	2.4	6.4

Ndff = N derived from fertilizer.

**Table 3:** Potential strategies to enhance the fertilizer N use efficiency by fruit trees.

Strategy	Mechanisms to Increase Tree N Use Efficiency
N fertilization practices	Split mineral N fertilizer rate into several applications possibly by fertigation; targeted supply of N in the soil volume explored by roots; application of N in the period of active root growth; promote internal N storage and remobilization also by foliar N applications; use organic N fertilizers.
Soil management	Promote the presence of legume plants among the orchard floor vegetation; use cover crops and incorporate into the soil their biomass; enhance the soil organic matter and the organic soil N, e.g., manure and compost, leave plant residues and mowed grasses on the orchard floor; promote presence of mycorrhizae that expand absorption ability and may allow organic N uptake.
Plant genotype	Choice of efficient plant genotypes with limited growth and high yield potential; rootstocks with a high root density, able to take up N at high rates; genotypes able to maximize photosynthesis with low leaf N.

**Table 4:** Agronomic and physiological conditions that improve the effectiveness of foliar N fertilizers (Source: adapted from [60]).

Foliar N Fertilizer Supplied to	Example
prevent or treat temporary N deficiency	After remobilisation has finished in spring and root N uptake is still low; latter, during fruit set/maturation, in N deficient plants
overcome limiting conditions of N availability or uptake	Poor root growth, low soil temperature, low soil moisture, poor soil aeration
increase reserve accumulation for remobilisation in the following year	In late summer-autumn
apply N to deep-rooted trees, when broadcasting is almost ineffective	When the soil supplied N does not reach the soil layers explored by roots due to the absence of rainfall or irrigation
